# Do job demands and resources differ between permanent and temporary eldercare workers in Sweden?

**DOI:** 10.1093/annweh/wxae077

**Published:** 2024-10-19

**Authors:** Nestor Lögdal, Sven Svensson, Jennie Jackson, Svend Erik Mathiassen, Gunnar Bergström, David M Hallman

**Affiliations:** Centre for Musculoskeletal Research, Department of Occupational Health, Psychology, and Sports Sciences, University of Gävle, Kungsbäcksvägen 47, 802 67 Gävle, Sweden; Centre for Musculoskeletal Research, Department of Occupational Health, Psychology, and Sports Sciences, University of Gävle, Kungsbäcksvägen 47, 802 67 Gävle, Sweden; Centre for Musculoskeletal Research, Department of Occupational Health, Psychology, and Sports Sciences, University of Gävle, Kungsbäcksvägen 47, 802 67 Gävle, Sweden; Centre for Musculoskeletal Research, Department of Occupational Health, Psychology, and Sports Sciences, University of Gävle, Kungsbäcksvägen 47, 802 67 Gävle, Sweden; Centre for Musculoskeletal Research, Department of Occupational Health, Psychology, and Sports Sciences, University of Gävle, Kungsbäcksvägen 47, 802 67 Gävle, Sweden; Unit of Intervention and Implementation Research for Worker Health, Institute of Environmental Medicine, Karolinska Institutet, Box 210, 171 77, Stockholm, Sweden; Centre for Musculoskeletal Research, Department of Occupational Health, Psychology, and Sports Sciences, University of Gävle, Kungsbäcksvägen 47, 802 67 Gävle, Sweden

**Keywords:** work environment conditions, physical, psychosocial, workload, support, influence, employment form, zero-hours contract

## Abstract

**Introduction:**

Eldercare organizations face high sickness absence rates and staff turnover and rely heavily on temporary workers to fill staffing gaps. Temporary workers may experience differences in job demands and resources compared with permanent workers, but this has been largely understudied.

**Objective:**

To compare perceived job demands and resources between permanent and temporary Swedish eldercare workers.

**Methods:**

Permanent and temporary eldercare workers in a Swedish municipality were invited to answer a digital survey on work environment conditions. Differences between permanent and temporary workers in job demands and resources were analyzed using multivariate analysis of variance adjusted for age, sex, place of birth, and percent of full-time work and univariate analyses were conducted to consider differences in specific factors.

**Results:**

A total of 1076 permanent and 675 temporary workers received the survey, and the final study sample included 451 permanent and 151 temporary workers. Multivariate analyses revealed that temporary workers reported statistically significant lower job demands compared to permanent workers, but no statistically significant differences in resources were found between the groups. Univariate analyses showed that temporary workers reported lower quantitative demands, perceived exertion, and time spent bending forward, than permanent workers. These data suggest comparable support across groups, but a higher workload among permanent workers.

**Conclusion:**

Our findings indicate that temporary workers experienced lower job demands than permanent workers, but that no notable difference was found in resources. Interventions aimed at distributing job demands more evenly among eldercare workers with different employment forms may be necessary.

**What’s Important About This Paper**

Temporary workers are common in eldercare, yet few studies have considered their work environment conditions. This study compares job demands and resources between permanent workers and temporary workers in eldercare and found that temporary workers experienced comparable resources, but lower job demands than permanent workers. Understanding differences in the work environment between permanent and temporary workers is necessary to guide future interventions to ensure sustainable working conditions for all workers.

## Introduction

Eldercare organizations face staffing shortages, which can be attributed in part to high rates of sickness absence (Swedish Association of Local Authorities and Regions 2017; OECD 2020) and worker turnover (Drennan and Ross 2019; Jurij et al. 2023). Both staffing shortages and fast staff turnover in eldercare have led to a large reliance on temporary workers (Ravalier et al., 2017; Swedish Association of Local Authorities and Regions, 2017). Temporary work arrangements, like casual work, fixed-term employment, or zero-hours contracts are often associated with higher job insecurity and lower income (Jonsson et al. 2019). However, temporary work can also give advantages such as greater flexibility in scheduling and increased control over working hours and leisure time (Casey and Alach 2004; Avram 2020). In Sweden, temporary workers without defined working hours (“Timvikarie” in Swedish and “Temp workers” from here on) comprise 28% of the eldercare workforce (Swedish Agency for Health and Care Services Analysis, 2021). These workers function as on-call employees who are summoned to work when needed and paid only for the hours worked (Swedish Association of Local Authorities and Regions, 2017; Statistics Sweden, 2020).

Poor work environment conditions in eldercare, including high physical (Januario et al. 2019, 2020, 2023), and psychosocial (Bratt and Gautun 2018; Januario et al. 2019; Stevens et al. 2022) demands, as well as limited resources, including lack of influence and workplace support (Peters et al. 2018; Jurij et al. 2023; Krsnik and Erjavec 2023) are likely contributors to the staffing shortages. Demands and resources in the work environment can be understood in terms of the Job Demands-Resources (JD-R) model (Bakker and Demerouti 2007). Demands can be physical, psychological, social, or organizational aspects of the job that require sustained physical, cognitive, and/or emotional efforts, and are therefore associated with strain to the worker (Bakker et al. 2022). Resources can also be physical, psychological, social, or organizational, but contrary to demands, resources support and motivate workers, foster learning, and personal growth, and may therefore reduce strain and potentially mitigate the effects of excessive demands (Bakker et al. 2022).

Despite the high prevalence of Temp workers in Swedish eldercare, there is a lack of research on whether their experiences of demands and resources at work differ from those of permanent employees. Internationally, such research is also scarce, and findings have been inconsistent. In a sample of homecare workers from the United Kingdom, similar job demands and resources were found among permanent and Temp workers (Ravalier et al. 2017). While in a later study, the research group found Temp homecare workers described low resources, including a power imbalance with managers that led Temp workers to feel pressured to accept all shifts they were offered, which in turn resulted in a perceived lack of control over their working hours (Ravalier et al. 2019). Concern over not being offered shifts could even lead Temp workers to feel compelled to accept more physically demanding, repetitive, or uncomfortable tasks which could further increase the risk for negative occupational health outcomes. Studies of Temp workers in other sectors, such as construction, materials handling, and assembly, have shown Temp workers perceive higher physical demands including more work done in awkward and static postures and under stricter time constraints than permanent workers (Kompier et al. 2009; Roquelaure et al. 2012). Further, Temp workers in health care, construction, and education have reported lower resources including lower levels of influence and less support from both supervisors and colleagues compared to permanent workers (Aronsson et al. 2002). Consideration of work organization and work environment conditions of all workers is crucial for establishing sustainable working environments and workplace equality. Understanding how Temp workers perceive their job demands and resources compared to permanent workers is a key first step to identifying potential inequalities (Eichhorst and Kalleberg 2023). Thus, the aim of this study was to compare perceived job demands and resources between permanent workers and Temp workers in Swedish eldercare, based on data from a mid-sized Swedish municipality.

## Methods

### Study design and population

**Fig. 1.**
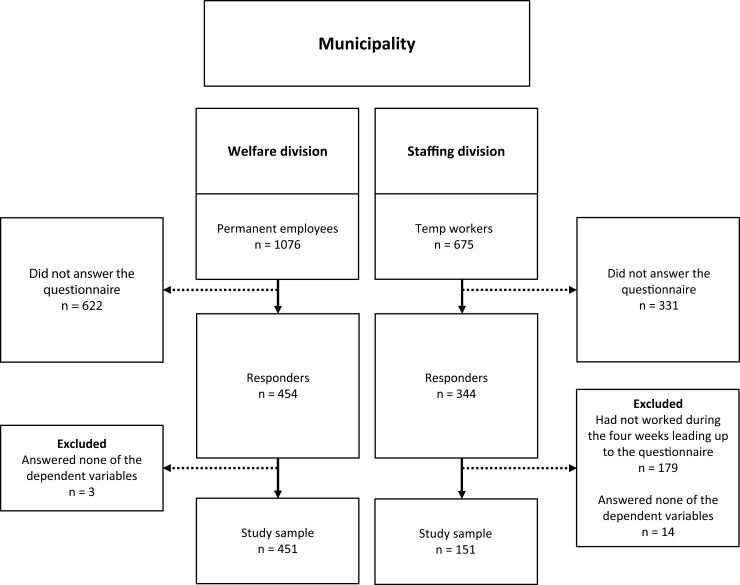
Structure of the organization and participant responses and dropouts.

This cross-sectional study recruited eldercare workers from 34 public units, including 20 nursing homes and 14 homecare units in the municipality of Gävle, Sweden. Gävle is Sweden’s 18th largest municipality with approximately 103,000 inhabitants. In Sweden, eldercare is the responsibility of the municipalities and can be offered both by private and public providers (Swedish Agency for Health and Care Services Analysis, 2021). Care is broadly divided between institutional care in nursing homes and homecare (Swedish Association of Local Authorities and Regions, 2020). Residents in these forms of care differ, with nursing homes typically accommodating older individuals with higher care needs compared to homecare (Swedish Association of Local Authorities and Regions, 2020). Workers in Swedish nursing homes tend to care for an average of 10.1 residents per shift, while homecare workers see an average of 11.8 residents (Szebehely et al. 2017; Szebehely 2020). The permanent and Temp workers were recruited from two separate organizational divisions: Welfare and Staffing (Figure 1). Both divisions agreed to participate and supplied workers’ contact information. Between November 2022 and April 2023, we distributed personal links to a questionnaire (Qualtrics XM© software, Qualtrics, Provo, UT) to 1076 permanent workers via their work e-mail and to 675 Temp workers via text messages to their private cell phones. Out of the permanent workers, 428 worked in homecare, and 648 in nursing homes, while 258 of the Temp workers worked in homecare, 348 in nursing homes, and 69 in both settings. Because some Temp workers worked in both homecare and nursing homes, we combined data across homecare and nursing homes. The study was approved by the Swedish Ethical Review Authority (Ref. no. 2019–06220).

### Eligibility criteria

Information about employment form (permanent or Temp worker) was provided by the organization for all workers prior to the administration of the survey. Eligible permanent workers were defined as unlicensed staff (i.e. assistant nurses and nurse’s aides) working with direct care in either homecare or nursing homes on a permanent contract (“tillsvidareanställning” in Swedish). Temp workers were eligible if they were registered in the municipality’s database of Temp workers and had worked in homecare, nursing homes, or both in the last year leading up to data collection, with access to information on their accumulated working hours over the year. Participants from both groups were included in the study if they had complete data on their date of birth, sex, and, for permanent workers, the percentage of full-time work. These variables were provided by the municipality. Participants also needed to have responded to questions addressing at least one of the dependent variables, i.e. job demands and resources (below). Additionally, Temp workers had to have worked at least one shift within the 4 wk preceding their response to the questionnaire to be included in the study.

### Job demands and resources

We measured both psychosocial and physical aspects of job demands using answers in the questionnaire to quantitative demands, perceived exertion, time in forward bending, and frequency of heavy lifting, all of which are known risk factors for sickness absence (Lund et al. 2006; Andersen et al. 2012; Holtermann et al. 2013; Stevens et al. 2022). Job resources were assessed by perceived social support from supervisors and colleagues, and influence at work. The two social support variables were chosen because poor support and workplace relationships have been cited as reasons for leaving the job (Clausen et al. 2014). Influence at work was chosen because it has the potential to reduce job strain during high demands (Schmidt and Diestel 2011).

Quantitative demands were measured using three items (e.g. *How often do you not have time to complete all your work tasks?*) from the third Copenhagen Psychosocial Questionnaire (COPSOQ III) (Berthelsen et al. 2020). The response scale had five points labeled: *Always*, *Often*, *Sometimes*, *Seldom*, *Never/hardly ever*, with the scale range 0-100. Perceived exertion was rated using the question *How physically straining do you normally consider your work to be?* (Borg 1998). The response scale ranged from 0 to 10, where 0 = *Not at all*, and 10 = *Extremely*. Time in forward bending was measured using the question *How large part of a typical working day do you work with your back bent forward?* with the four-point response scale: 1 = *Almost never*, 2 = *5–30 min/day*, 3 = *31–60 min/day*, 4 = *More than 60 min/day* (modified from Wiktorin et al. 1999). The number of heavy lifts per day was measured using the question *Do you lift or carry heavy objects (more than 10 kg) at work*?, with the four-point response scale: 1 = *Almost never*, 2 = *1–5 times/day*, 3 = *6–10 times/day*, 4 = *More than 10 times/day* (modified from Wiktorin et al. 1999).

Job resources were assessed using the dimensions Influence at Work (four items, e.g. *Can you influence the amount of work assigned to you?*), Social Support from the immediate Supervisor (single item, *How often do you get help and support from your immediate superior, if needed?*), and Social Support from Colleagues (single item, *How often do you get help and support from your colleagues, if needed?*), all from the Swedish COPSOQ III (Berthelsen et al. 2020). All questions were answered on five-point scales with range 0*–*100. The response labels for Influence at work and the two support questions were *Always*, *Often*, *Sometimes*, *Seldom*, *Never/hardly ever*.

For Quantitative demands and Influence at Work, indices were computed as each participant’s mean score across the individual items (Cronbachs α = 0.74, for both indices). For an index to be computed, the respondent had to have answered at least half of the items.

### Demographic and personal data

Demographic and personal information included: highest completed education level (*elementary school*, *high school*, *secondary school that is not college/university*, *or college/university*), place of birth (*Sweden*, *not Sweden*), marital status (*married/registered partner/cohabitants*, *single household*, *not sure*), if the workers had children of any ages living at home (*Yes*, *No*), and self-rated health measured using the question *In general, would you say your health is…* from the 36 Item Short Form Survey (SF-36) (Ware Jr. 1999), with the five-point response scale: *Poor*, *Fair*, *Good*, *Very good*, and *Excellent*, respectively.

### Covariates

Date of birth, sex, place of birth, and percent of fulltime work (permanent workers) or accumulated working hours in the previous year (Temp workers) were used as covariates. From the accumulated working hours over the year, an average percent of fulltime work was computed for the Temp workers by dividing their accumulated working hours by the number of fulltime working hours in the year (excluding bank holidays and weekends).

### Statistical analyses

All data processing and all statistical analyses were conducted in R (R Core Team 2023) using the tidyverse suite of packages (Wickham et al. 2019) and the psych package (Procedures 2023). Data are presented as counts, proportions, means, and standard deviations (SD).

### Coverage analysis

To examine differences between responders and non-responders, we performed a coverage analysis comparing age, working time, and proportion of men and women. For the continuous variables age and percent of fulltime work, differences were assessed using unequal variances t-tests (also known as Welchs *t*-test), while differences in the proportion of men and women were tested using chi-squared tests.

### Comparisons of demands and resources between Temp and permanent workers

We investigated differences in job demands and resources between permanent and Temp workers using unadjusted multivariate analysis of variance (MANOVA) followed by MANOVAs adjusted for covariates. These models were run separately for demands and resources. Demands included the dependent variables: quantitative demands, rating of perceived exertion, time in forward bending, and frequency of heavy lifting. Resources included the dependent variables: influence, social support from supervisors, and social support from colleagues. Statistically significant differences in the multivariate sample means were followed up with unadjusted and adjusted univariate analyses of variance (ANOVA) performed on each individual demand and resource variable.

To check the robustness of the results, the primary analyses were followed by two sensitivity analyses. In the first, we adjusted the models for self-rated health in addition to the initial adjustments. In the second, we made mutual adjustments between the demand and resource variables (i.e. models analyzing demands were adjusted for resources, and vice versa).

The dependent variables showed no severe outliers or multicollinearity, and the residuals showed no pronounced deviations from a normal distribution. However, some heteroscedasticity was observed in the scale location and residuals versus fits plots, and the heteroscedasticity was also confirmed by Levene’s test. Traditional multivariate analyses of variance can be sensitive to unequal sizes of groups with different variances, which may lead to elevated rates of type I errors (Finch 2005), and we conducted robustness checks on our unadjusted models using non-parametric methods in the npmv package (Burchett et al., 2017). The non-parametric tests showed similar results as the parametric tests: and since non-parametric tests do not allow for covariates, we continued with parametric tests. We used partial eta squared (ηp^2^) as a standardized effect size measure with small (0.01), medium (0.06), and large (0.14) effect sizes defined according to Cohens recommendation (Cohen 1988). The α level was set to 0.05.

### Sensitivity analyses

#### Results

Out of the 1076 eligible permanent workers who received the questionnaire, 454 responded (Fig. 1). Of these, three workers did not give responses for any of the dependent variables and were excluded. Thus, data from 451 permanent workers were included, resulting in a response rate of 41.9%. Of the 675 Temp workers who received the questionnaire, 344 responded. Of these, 179 had not worked any shift within the 4 wk prior to responding to the questionnaire, and an additional 14 did not give responses for any of the dependent variables: all these 193 workers were excluded. Thus, data from 151 Temp workers were included, resulting in a response rate of approximately 46% among those who had worked within the latest 4 wk (Fig. 1).

### Coverage

In both groups, respondents were older and had a higher percent working time than non-responders with age and working time differences for permanent (*t* (1030.1) = 2.74, *P* = 0.006: *t* (1009.6) = 3.1, *P* = 0.002) and Temp workers (*t* (243.17) = 2.7, *P* = 0.009: *t* (222.3) = 3.2, *P* = 0.002) all being significant. No statistically significant differences were found in the ratio of males-to-females between responders and non-responders for permanent workers (χ^2^ (1) = 2.2, *P* = 0.11) or for Temp workers (χ^2^ (1) = 2.2, *P* = 0.14).

### Demographic data

Demographic data for both permanent and Temp workers included in the analyses are presented in Table 1. On average, the Temp workers were younger, worked less, and comprised a higher proportion of males and workers born outside of Sweden than the group of permanent workers. Moreover, Temp workers also rated their health as better than the permanent workers.

**Table 1.** Demographic data for permanent and Temp workerst.

Variable	Permanent workers	Temp workers
**Age Mean (SD)**	49.4 (10.5)	38.5 (16.4)
**Sex *n* (%)**		
Men	62 (13.7)	47 (31.1)
Women	389 (86.3)	104 (68.9)
**Eldercare setting *n* (%)**		
Homecare	188 (41.7)	52 (34.4)
Nursing home	263 (58.3)	81 (53.6)
Mixed	0 (0)	18 (11.9)
**Tenure in years Mean (SD)**	17 (12.6)	3.8 (3.5)
**Percent of fulltime work (%) Mean (SD)**	93.6 (11.5)	26.9 (20.9)
**Number of shifts previous year Mean (SD)**	Not available	81.4 (63.2)
**Self-rated health Mean (SD)** ^a^	2.9 (0.9)	3.5 (1.0)
**Education *n* (%)**		
Elementary school	49 (10.9)	23 (15.2)
High school	225 (49.9)	67 (44.4)
Secondary school (not college/university)	141 (31.3)	29 (19.2)
College/university	32 (7.1)	28 (18.5)
Missing	4 (0.9)	4 (2.6)
**Born in Sweden *n* (%)**		
Yes	285 (63.2)	47 (30.9)
No	160 (35.5)	96 (63.8)
Missing	6 (1.3)	8 (5.3)
**Marital status *n* (%)**		
Married/registered partner/cohabitant	280 (62.1)	62 (41.1)
Single household	161 (35.7)	67 (44.4)
Not sure	5 (1.1)	10 (6.6)
Missing	5 (1.1)	12 (7.9)
**Workers with children at home (%)**		
Yes	243 (53.9)	91 (60.3)
No	208 (46.1)	60 (39.7)

^a^19 missing responses in the permanent worker group and 76 missing responses in the Temp worker group.

### Differences in job demands and resources between Temp and permanent workers

The mean values for the variables representing demands and resources are presented in Table 2. The unadjusted multivariate analysis showed a statistically significant difference in perceived job demands between the Temp and permanent worker groups (*F*(4, 545) = 10.6, *P* < 0.001, ηp^2^ = 0.07, medium effect size), and the effect remained after adjusting for covariates (*F*(4, 530) = 9.9, *P* < 0.001, ηp^2^ = 0.07). The adjusted ANOVAs showed that Temp workers reported significantly lower values for quantitative demands, perceived exertion, and time in forward bending compared with the permanent workers (Table 3).

**Table 2.** Job demands and resources for permanent and temporary workers.

Variable	Permanent workers		Temp workers		
Demands	Mean (SD)	*n* (%)	Mean (SD)	*n* (%)	Scale range
Quantitative demands	41.8 (20.1)	451 (100)	34.9 (24.0)	151 (100)	0–100
Rating of perceived exertion	5.9 (2.4)	443 (98.2)	4.4 (3.0)	114 (75.5)	0–10
Forward bending	2.8 (1.0)	445 (98.7)	2.3 (1.0)	119 (78.8)	1–4
Heavy lifting	2.3 (1.0)	446 (98.7)	2.0 (0.9)	120 (78.8)	1–4
**Resources**					
Influence	36.9 (21.6)	448 (99.3)	41.2 (24.8)	148 (98.0)	0–100
Social support from supervisor	69.9 (27.8)	449 (99.6)	66.6 (33.2)	134 (88.7)	0–100
Social support from colleagues	78.6 (20.0)	446 (98.9)	79.1 (24.3)	133 (88.1)	0–100

**Table 3.** Results from the unadjusted (left) and adjusted (right) univariate analyses of variance (ANOVA). Statistically significant differences are marked in bold text.

	Unadjusted univariate comparisons				Adjusted univariate comparisons^a^			
Variable	DF	*F*	*P*	ηp2	DF	*F*	*P*	ηp2
**Demands**								
Quantitative demands	1, 600	12.0	**0.001**	**0.02**	1, 582	11.2	**0.001**	**0.02**
Rating of perceived exertion	1, 551	30.8	**<0.001**	**0.05**	1, 536	24.5	**<0.001**	**0.04**
Forward bending	1, 558	24.9	**<0.001**	**0.04**	1, 541	25.0	**<0.001**	**0.04**
Heavy lifting	1, 558	6.4	**0.012**	**0.01**	1, 541	3.4	0.065	0.01
**Resources**								
Influence	1, 593	4.0	**0.047**	**0.01**	1, 575	3.5	0.061	0.01
Social support from supervisor	1, 578	1.7	0.191	<0.01	1, 562	1.8	0.183	0.00
Social support from colleagues	1, 574	0.1	0.773	<0.01	1, 557	0.01	0.918	0.00

^a^Adjusted for date of birth, sex, place of birth (Sweden/not Sweden), and percent of fulltime work.

DF = degrees of freedom; ηp2 = partial eta squared.

For job resources, neither the unadjusted nor the adjusted multivariate analyses showed statistically significant differences between the Temp and permanent workers, and the effect sizes were small (*F*(3, 564) = 2.0, *P* = 0.11, ηp^2^ = 0.01) and (*F*(3, 548) = 1.7, *P* = 0.16, ηp^2^ = 0.01), respectively. In the unadjusted univariate models, influence at work was significantly higher for Temps compared to permanent workers, but even this effect size was small (Table 3). Although the effect size did not change in the adjusted model, it lost its statistical significance (Table 3).

The sensitivity analyses for the multivariate models are presented in Supplementary Table 1, and the univariate analyses are shown in Supplementary Table 2. For job demands, the additional adjustment for self-rated health (Model 1) and the adjustment for job resources (Model 2) led to results that were, in general, consistent with the primary analysis in terms of statistical significance and effect sizes. For job resources, the additional adjustment for self-rated health produced results similar to the primary analysis, though with a slight reduction in *P*-values, resulting in statistical significance for influence at work (Supplementary Table 1). However, we found no statistically significant differences in resources between the groups when adjusting for job demands.

## Discussion

The aim of this study was to compare perceived job demands and resources between Temp workers and permanent workers in eldercare. Our study examined 451 permanent workers and 151 Temp workers from a mid-sized municipality in mid-Sweden. We found that Temp workers reported lower demands, including lower quantitative demands, perceived exertion, frequency of forward bending, and fewer heavy lifts compared to permanent workers, but no notable differences were found between the two groups in terms of resources, represented by influence at the workplace, support from supervisor, and support from colleagues. These findings suggest comparable experiences of support across groups, but an unevenly distributed workload, with permanent workers having a larger workload.

To our knowledge, very few studies have investigated job demands and resources in permanent and Temp workers in eldercare. One survey from the United Kingdom found similar job demands and resources between permanent and Temp workers (Ravalier et al. 2017), while a later study based on interviews indicated that Temp workers perceive limited resources and a lack of control over working hours (Ravalier et al. 2019). Our results, however, show that Temp workers in Swedish eldercare experience favorable demands and have comparable resources to permanent workers.

Several factors may explain the observed differences in how demands are perceived. Permanent and Temp workers differed in accumulated working hours in the previous year. We accounted for this difference by including it as a covariate in our adjusted analyses, which did not change the result. It, therefore, seems unlikely that the differences in demands were attributed to fewer workdays for Temp workers. However, the distribution of working hours over the year may differ between the groups. Temp workers may have more opportunities for rest and recovery between shifts, which our covariate may not account for. Another possible explanation for the lower demands in Temp workers could be differences in responsibility and assigned tasks compared to permanent workers. With 45% of Temp workers in eldercare lacking formal education and training in care (Swedish National Board of Health and Welfare, 2015), this educational gap may result in Temps performing different tasks or being restricted from certain responsibilities, which the permanent staff will then have to take. Consequently, Temp workers may not fully compensate for the permanent staff they replace, leading permanent workers to perform more demanding tasks and/or assist with Temp workers’ tasks on top of their regular duties. This may create uneven workloads in the two groups. This explanation agrees with a study conducted in London hospitals, finding that permanent workers perceived less experienced Temp workers as a distraction, who required guidance and management from the permanent staff (Bajorek and Guest 2019). Overall, the finding of lower demands in Temp workers was consistent when adjusting for our initial covariates, and it remained robust in the sensitivity analyses with additional adjustment for self-rated health and job resources.

The Temp workers rated their perceived quantitative demands approximately seven points lower than the 41.8 rated by the permanent workers, which is considered a clinically meaningful difference (Pejtersen et al. 2010). The mean value of quantitative demands for permanent workers in the current study is slightly lower than the 44.5 reported in Danish eldercare workers (Stevens et al. 2022). Similarly, Temp workers reported lower perceived exertion compared to permanent workers (4.4 versus 5.0), and the permanent workers rated even their perceived exertion slightly lower than Danish eldercare workers, whose exertion levels ranged between 6.4 and 6.9 (Rasmussen et al. 2015; Karstad et al. 2018; Januario et al. 2019, 2020). Regarding job resources, we found a small but statistically significant difference between the groups, with temporary workers reporting slightly higher levels of influence. This finding agrees with results reported in (Ravalier et al. 2017) and may be explained by Temp worker’s leeway to accept or decline work shifts, which could increase their sense of influence at work. However, this result should be interpreted with caution, as it was not consistent across the adjusted models. The statistical significance decreased in the adjusted primary model compared to the unadjusted model, increased in the first sensitivity analysis when adjusting for health, and disappeared in the second sensitivity analysis when adjusting for job demands (Table 3, Supplementary Tables 1 and 2). This pattern makes it difficult to determine whether the difference reflects a true effect or is influenced by confounding factors. Nonetheless, the difference was very small and likely of limited practical relevance.

### Strengths and limitations

The main strength of this study is that we obtained reliable information from organizational records on employment forms and working time for both permanent and Temp workers. Despite the differences in age, proportion of men and women, employment rate, and country of birth between the groups, we observed only marginal differences between the unadjusted and adjusted models. This suggests that the observed differences in demands and resources between the groups are not primarily attributable to demographic factors. The study suffers some limitations. The data were collected from a single mid-size municipality in Sweden, which may limit the generalizability of the findings to eldercare organizations in municipalities of different sizes and in other countries with varying working conditions. We analyzed the groups without considering whether they worked in homecare or nursing homes, as some Temp workers were employed in both settings. This approach may have influenced our results due to differences in work structure and the characteristics of the people cared for in each setting. For example, homecare workers often work alone and have less access to assistive devices (Wipfli et al. 2012) while nursing home caretakers generally care for individuals with greater needs than those receiving homecare (Schön et al. 2016). Other aspects to consider are the relatively small sample size and rather small coverage of the Temp workers. The coverage analysis indicated some slight differences between responders and non-responders in age and working time, which may limit generalizability to younger Temp workers with few working hours. Lastly, all variables in the current study are self-reported. Self-reported measures, particularly those involving physical work exposures are prone to bias and imprecision (Prince et al. 2008), which may have influenced our results for time spent bent forward and frequency of heavy lifting.

### Future studies and practical applications

Our findings highlight the need for future studies to investigate the types of work tasks assigned to permanent and Temp workers, and how these tasks are distributed. Such research is essential for developing and evaluating interventions aimed at distributing work demands more evenly among eldercare workers with different employment forms. Examples of strategies could be to assign specific Temp workers to specific units whenever there is a staffing gap, or alternatively, to maintain individual pools of Temp workers for each unit. Such interventions would ensure that units are staffed with workers familiar with the routines. Additionally, offering training for Temp workers to establish a baseline level of skills could be a potential strategy to alleviate the workload for permanent workers. Other important areas for future research are the differences in work structure between homecare and nursing homes, and whether these result in different exposures. Distinct exposures may necessitate tailored interventions for each group of workers, and findings from one setting might not be generalizable to the other. Finally, we recommend future longitudinal studies to address the relationships between employment forms, work environment, and health in the eldercare sector.

## Conclusion

This study examined differences in job demands and resources between permanent and Temp workers using data from eldercare in a Swedish—municipality. While the two groups experienced comparable resources, our findings suggest notable differences in job demands, with permanent workers reporting higher demands than Temp workers. To ensure a sustainable working environment that can promote health for all workers, interventions aimed at distributing job demands more evenly among eldercare workers with different employment forms may be necessary.

## Supplementary material

Supplementary material is available at *Annals of Work Exposures and Health* online.

wxae077_suppl_Supplementary_Tables_1-2

## Data Availability

The data used are available on request.
