# Supplementary material for: Do job demands and resources differ between permanent and temporary eldercare workers in Sweden?
Source: Ann Work Expo Health. 2024 Oct 19;69(1):71–80. doi: 10.1093/annweh/wxae077 (PMC11706790; doi:10.1093/annweh/wxae077)
Supplement: wxae077_suppl_Supplementary_Tables_1-2 [file wxae077_suppl_supplementary_tables_1-2.docx]

**Title:** Do job demands and resources differ between permanent and temporary eldercare workers in Sweden?

Nestor Lögdal ^1^, Sven Svensson ^1^, Jennie Jackson ^1^, Svend Erik Mathiassen ^1^, Gunnar Bergström ^1, 2^, David M Hallman

^1^ Centre for Musculoskeletal Research, Department of Occupational Health, Psychology, and Sports Sciences, University of Gävle, Kungsbäcksvägen 47, 802 67, Gävle, Sweden

^2^ Unit of Intervention and Implementation Research for Worker Health, Institute of Environmental Medicine, Karolinska Institutet, Box 210, 171 77, Stockholm, Sweden

**Table 1. Supplementary file.** Results from the multivariate sensitivity analyses, adjustments explained in table footnotes.

|  | **Multivariate comparisons Model 1** | | | | **Multivariate comparisons Model 2** | | | |  |  |  |  |  |  |
| --- | --- | --- | --- | --- | --- | --- | --- | --- | --- | --- | --- | --- | --- | --- |
| **Variable** | **DF** | **F** | **p** | **ηp2** | **DF** | **F** | **p** | **ηp2** |  |  |  |  |  |  |
| Demands | 4, 482 | 8.3 | **< 0.001** | **0.06** | 4, 509 | 9.8 | **< 0.001** | **0.07** |  |  |  |  |  |  |
| Resources | 3, 477 | 2.7 | **0.048** | **0.02** | 3, 509 | 1.1 | 0.37 | 0.01 |  |  |  |  |  |  |
| Model 1 adjusted for date of birth, sex, place of birth, self-rated health, and percent of fulltime work | | | | | | | | |  |  |  |  |  |  |
| Model 2 adjusted for date of birth, sex, place of birth, percent of fulltime work, and mutual adjustments between demands and resources | | | | | | | | | | | | | | |
| DF = degrees of freedom | | | | | | | | | | |  |  |  |  |

**Table 2. Supplementary file.** Results from the univariate sensitivity analyses, adjustments explained in table footnotes.

|  | **Univariate comparisons Model 1** | | | | **Univariate comparisons Model 2** | | | |
| --- | --- | --- | --- | --- | --- | --- | --- | --- |
|  |  |  |  |  |  |  |  |  |
| **Variable** | **DF** | **F** | **p** | **ηp2** | **DF** | **F** | **p** | **ηp2** |
| **Demands** |  | | | |  | | | |
| Quantitative demands | 1, 492 | 10.4 | **0.001** | **0.02** | 1, 545 | 13.1 | **< 0.001** | **0.02** |
| Rating of perceived exertion | 1, 488 | 15.3 | **< 0.001** | **0.03** | 1, 516 | 26.6 | **< 0.001** | **0.05** |
| Forward bending | 1, 488 | 20.0 | **< 0.001** | **0.04** | 1, 522 | 20.8 | **< 0.001** | **0.04** |
| Heavy lifting | 1, 488 | 0.6 | 0.439 | 0.00 | 1, 522 | 2.3 | 0.13 | 0.00 |
| **Resources** |  |  |  |  |  |  |  |  |
| Influence | 1, 487 | 8.1 | **0.005** | **0.02** | 1, 522 | 1.1 | 0.29 | 0.00 |
| Social support from supervisor | 1, 489 | 0.1 | 0.794 | 0.00 | 1, 521 | 1.3 | 0.26 | 0.00 |
| Social support from colleagues | 1, 482 | 0.0 | 0.998 | 0.00 | 1, 517 | 0.01 | 0.93 | 0.00 |
| Model 1 adjusted for date of birth, sex, place of birth, self-rated health, and percent of fulltime work | | | | | | | | |
| Model 2 adjusted for date of birth, sex, place of birth, percent of fulltime work, and mutual adjustments between demands and resources | | | | | | | |  |
| DF = degrees of freedom | | | |  |  |  |  |  |
